# Using MEDLINE Elemental Similarity to Assist in the Article Screening Process for Systematic Reviews

**DOI:** 10.2196/medinform.3982

**Published:** 2015-08-31

**Authors:** Xiaonan Ji, Po-Yin Yen

**Affiliations:** ^1^ The Ohio State University Department of Biomedical Informatics Columbus, OH United States

**Keywords:** systematic review, evidence-based medicine, automatic document classification, relevance feedback

## Abstract

**Background:**

Systematic reviews and their implementation in practice provide high quality evidence for clinical practice but are both time and labor intensive due to the large number of articles. Automatic text classification has proven to be instrumental in identifying relevant articles for systematic reviews. Existing approaches use machine learning model training to generate classification algorithms for the article screening process but have limitations.

**Objective:**

We applied a network approach to assist in the article screening process for systematic reviews using predetermined article relationships (similarity). The article similarity metric is calculated using the MEDLINE elements title (TI), abstract (AB), medical subject heading (MH), author (AU), and publication type (PT). We used an article network to illustrate the concept of article relationships. Using the concept, each article can be modeled as a node in the network and the relationship between 2 articles is modeled as an edge connecting them. The purpose of our study was to use the article relationship to facilitate an interactive article recommendation process.

**Methods:**

We used 15 completed systematic reviews produced by the Drug Effectiveness Review Project and demonstrated the use of article networks to assist article recommendation. We evaluated the predictive performance of MEDLINE elements and compared our approach with existing machine learning model training approaches. The performance was measured by work saved over sampling at 95% recall (WSS95) and the F-measure (F_1_). We also used repeated analysis over variance and Hommel’s multiple comparison adjustment to demonstrate statistical evidence.

**Results:**

We found that although there is no significant difference across elements (except AU), TI and AB have better predictive capability in general. Collaborative elements bring performance improvement in both F_1_ and WSS95. With our approach, a simple combination of TI+AB+PT could achieve a WSS95 performance of 37%, which is competitive to traditional machine learning model training approaches (23%-41% WSS95).

**Conclusions:**

We demonstrated a new approach to assist in labor intensive systematic reviews. Predictive ability of different elements (both single and composited) was explored. Without using model training approaches, we established a generalizable method that can achieve a competitive performance.

## Introduction

Systematic reviews provide summaries of evidence from high quality studies to answer specific research questions. They are regularly used in health care [1-4] and for health policy making [5]. Evidence-based medicine (EBM) relies heavily on the use of synthesized, up-to-date research evidence to make decisions. Systematic reviews are considered the highest quality source of evidence for EBM [6]. However, systematic reviews require a series of very resource and time intensive steps [4] that typically require several months to complete [7]. Such workload and resource challenges can limit the tractability of an individual review, the ability to fund a review, and also the ability to respond to new evidence that may require an update to an existing review.

MEDLINE is a biomedical literature database that stores and indexes a variety of relevant publications and is a primary resource for identifying studies for systematic reviews targeting the health sciences. However, the size of MEDLINE increases at a rate of over 12,000 articles per week, including reports related to over 300 randomized trials [8]. The Cochrane Collaboration is an international organization dedicated to producing up-to-date systematic reviews, with more than 15,000 people participating in the work [9]. According to The Cochrane Collaboration, more than 10,000 systematic reviews are needed for existing effectiveness research [9]. In addition, a recent study reported that 23% of reviews require updates within 2 years [10]. With the need to conduct a large amount of original and updated systematic reviews, it is essential to improve the efficiency of producing systematic reviews and their incumbent synthesized knowledge.

A systematic review is commonly conducted by domain experts who are able to draft systematic review scopes, retrieve relevant citations, assess study quality, and synthesize evidence. The process can be broken down into 15 steps [11]. Expert reviewers first identify the systematic review scope and research questions, and then generate search strategies to explore related databases (eg, MEDLINE). The search result is a list of citations usually organized in reference management software (eg, EndNote, RefWorks). Before synthesizing relevant evidence, expert reviewers need to classify articles based on the title and abstract. Then through the article screening (or article selection) process, relevant articles will proceed to the full-text level. In most systematic reviews, expert reviewers include a small portion ranging from 2% to 30% of citations at the title and abstract level; 1.6% to 27% get included at the full-text level [7]. In other words, expert reviewers spend most of their effort excluding non-relevant or low quality studies. To accelerate this process, several machine learning approaches (ie, naive Bayes and support vector machine) [7,12-15] were proposed to focus on facilitating and enhancing the title and abstract level triage, abstracts screening [11], or article screening, which is crucial and time-consuming as it requires expert reviewers to screen a large amount of literature. The intelligent article selection process can be also called citation classification or citation screening.

In this paper, we proposed to use established and predetermined article relationships and incorporate the concept of active machine learning to iteratively recommend articles and receive feedback from human reviewers. Although the idea of integrating human judgment sounds similar to the active learning approach implemented in Wallace’s work [13,14], our approach uses a different strategy. We do not formulate a classification model. Instead, we generate an article network representing the relationships between articles. We use the articles classified by human reviewers as a reference set to recommend the next similar article. There is no model trained during the recommendation procedure. The approach is similar to relevance feedback, a feature in some information retrieval systems. In general, users classify documents as relevant or irrelevant and provide the feedback to the information retrieval system. The information retrieval system then uses this information to retrieve documents similar to the relevant documents. Relevance feedback is commonly used as an automatic technique for queries modification. The process of relevance feedback is executed as a cycle of activity that refines queries in each iteration of feedback collection [16,17].

**Figure 1:**
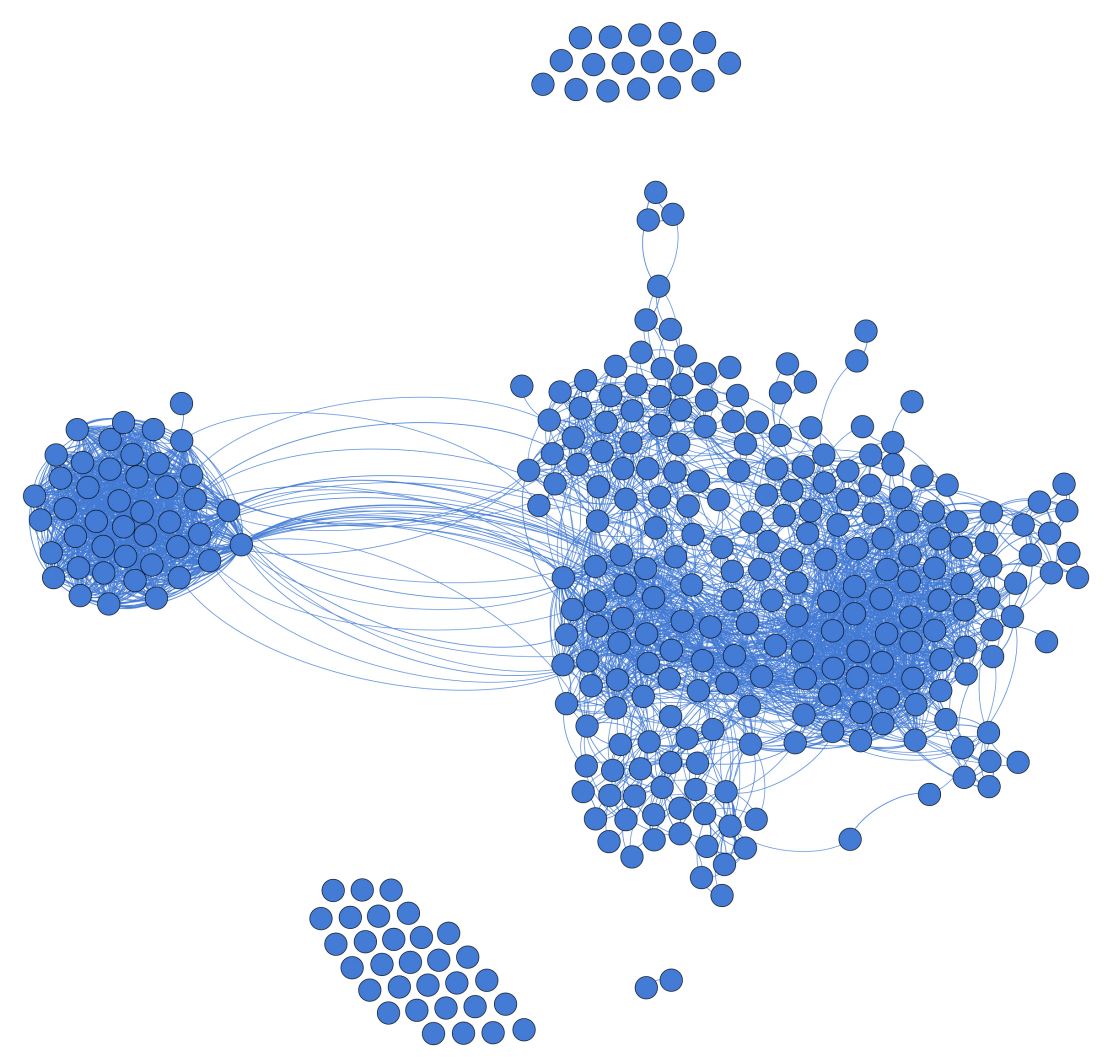
Illustrated article network.

The predetermined relationships between articles can be conceptualized as an article network, which is different from the traditional citation network. A traditional citation network uses the *citing* and *cited by* of an article to build the network [18]. We build article networks based on the similarity of any paired articles. Our similarity metric is calculated using data elements [19] from an article, such as title, abstract, medical subject heading, author, and publication type. Under this concept, each article is modeled as a node in the network and the relationship (similarity) between two articles is modeled as an edge connecting them. Although the network method is not novel in the general document clustering area, we are the first to use the approach to facilitate systematic reviews and demonstrate its strength. Figure 1 shows an illustrated network of a real systematic review (Urinary Incontinence) displayed in an aesthetically pleasing force-directed graphic layout. Theoretically, the network should be a complete graph in which every pair of articles has an edge representing the similarity between them. For visualization purposes, we eliminated the edges with lower similarity scores to provide a more human readable network.

During our preliminary experiments, we found that a similarity score composed of all MEDLINE elements does not work well for every systematic review. We suspected that some elements (eg, title, abstract, publication type, MeSH, author) are better predictors for recommendations than others. Therefore, the purpose of our study was to answer two research questions. When an article is classified as included, what element(s) are better to use to calculate the similarity score to predict the next relevant article? Since every element plays a different role and should be weighted accordingly, what combinations and weights of elements are better to predict the next relevant article?

## Methods

### Data Source

To evaluate our approach, we used 15 publicly available completed systematic review samples produced by the Drug Effectiveness Review Project (DERP) (coordinated by the Center for Evidence-Based Policy at Oregon Health and Science University) [20]. These 15 systematic reviews were completed by experienced and knowledgeable human expert reviewers, with inclusion and exclusion decisions made by at least two expert reviewers. Table 1 shows the number and percentage of articles included at abstract level decision and full-text level decision.

For instance, the review for ACE Inhibitors has a total of 2544 citations. Based on the abstracts, 183 (7.19%) were included; after full-text reading, 41 (1.61%) were included in the ACE Inhibitor systematic review. The final inclusion rates range from 0.78% to 27.04%. The 15 systematic reviews are also the same test collection previously used and made publicly available by Cohen et al [7]. Using the PubMed Identifier (PMID), we downloaded the full record in MEDLINE format [19] and extracted the data elements title, abstract, publication types, author and medical subject heading (MeSH) as the input.

**Table 1:** Total article numbers and rates of inclusion.

SR report topic	Total	Abstract n (%)	Full text n (%)
ACE inhibitors	2544	183 (7.19)	41(1.61)
ADHD	851	84 (9.87)	20 (2.35)
Antihistamines	310	92 (29.68)	16 (5.16)
Atypical antipsychotics	1120	363 (32.41)	146 (13.04)
Beta blockers	2072	302 (14.58)	42 (2.03)
Calcium channel blockers	1218	279 (22.91)	100 (8.21)
Estrogens	368	80 (21.74)	80 (21.74)
NSAIDS	393	88 (22.39)	41 (10.43)
Opioids	1915	48 (2.51)	15 (0.78)
Oral hypoglycemics	503	139 (27.63)	136 (27.04)
Proton pump inhibitors	1333	238 (17.85)	51 (3.83)
Skeletal muscle relaxants	1643	34 (2.07)	9 (0.55)
Statins	3465	173 (4.99)	85 (2.45)
Triptans	671	218 (32.49)	24 (3.58)
Urinary incontinence	327	78 (23.85)	40 (12.23)

### MEDLINE Elements

MEDLINE elements are the fields in the MEDLINE format that document the major pieces of information of a publication (article) [19]. The MEDLINE display format is used in PubMed MEDLINE records. As the most informative elements, title (TI), abstract (AB) and MeSH (MH) elements are widely used in related work to build feature spaces for machine learning algorithms. Publication type (PT) is also selected by some studies [15,21] as it could be a key factor for inclusion or exclusion decisions. In our preliminary work, we found that author information also has some predictive value in the article screening process. Therefore, in this study, in addition to TI, AB, MH, and PT, we also included the author (AU) element in our experiments.

### Similarity Score

We calculate the similarity score using cosine similarity [22]. Cosine similarity is widely applied to text mining and measures the cosine of the angle between a pair of vectors. It reflects the degree of similarity based on the presence and frequency of words or terms in each text. For every pair of AUs, PTs, and MHs, we simply compared them by exact string matching, because a minor difference may completely alter the outcome. For example, even if two author names are very similar, they may be two different people. However, TI and AB are free text. To calculate the similarity between two TIs and between two ABs, we preprocessed TIs and ABs by removing some common words from the PubMed stop word list [23] (eg, the, is, are) that appear frequently in text, stemming each word by the classic Porter Stemmer algorithm [24]. This approach, named alphabetic features, also has been verified to be an effective method to represent an article [25]. The resulting similarity score ranges from 0 to 1 for each element, where 0 indicates independence and 1 means exactly the same. In summary, the similarity score is the sum of the MEDLINE element similarities.

### Simulated Interactive Recommendation Process

#### Overview

In this study, there is no human reviewer in this experimental process. The interactive recommendation process is simulated using the 15 completed DERP systematic reviews.

**Figure 2:**
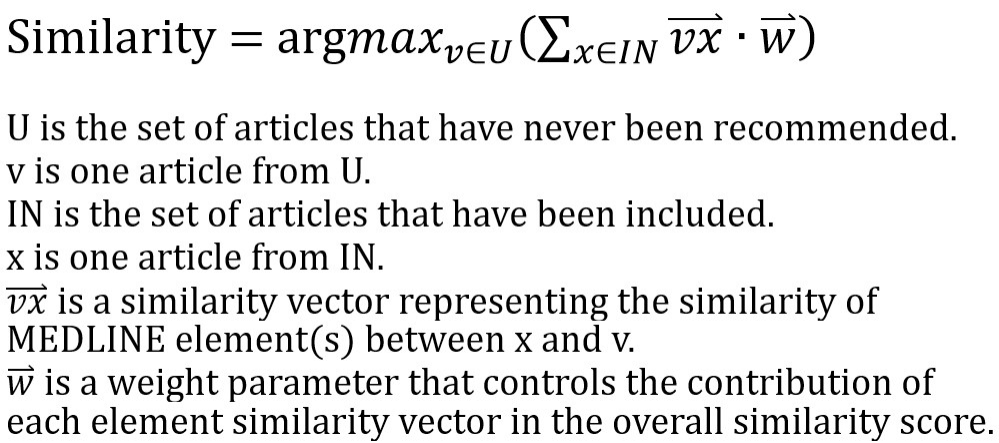
Calculation of the similarity between articles.

After identifying a list of articles to be screened for a systematic review, the recommendation process starts with calculating the similarity scores of any pairs of articles. This process constructs the relationship of the articles and builds a conceptualized article network. The first recommended article is selected based on key questions and search strategies of the systematic review. Once a recommended article is classified as included (IN) or excluded (EX), an IN list and an EX list are created (in this study, we used completed systematic reviews, which have predetermined decisions to simulate this step). We then iteratively recommend relevant articles based on the similarity to the IN. Assuming V is the set of all articles and U is the set of articles that have never been recommended, U is defined as U=V−IN−EX. Therefore, the sum of similarity scores represents the similarity between an article v with article(s) x in IN (see Figure 2).

In the formula, *vx* is a similarity vector representing the similarity of MEDLINE element(s) between x and v. The weight parameter *w* controls the contribution of each element similarity vector in the overall similarity score. We recommend articles with the highest overall similarity scores.

**Figure 3:**
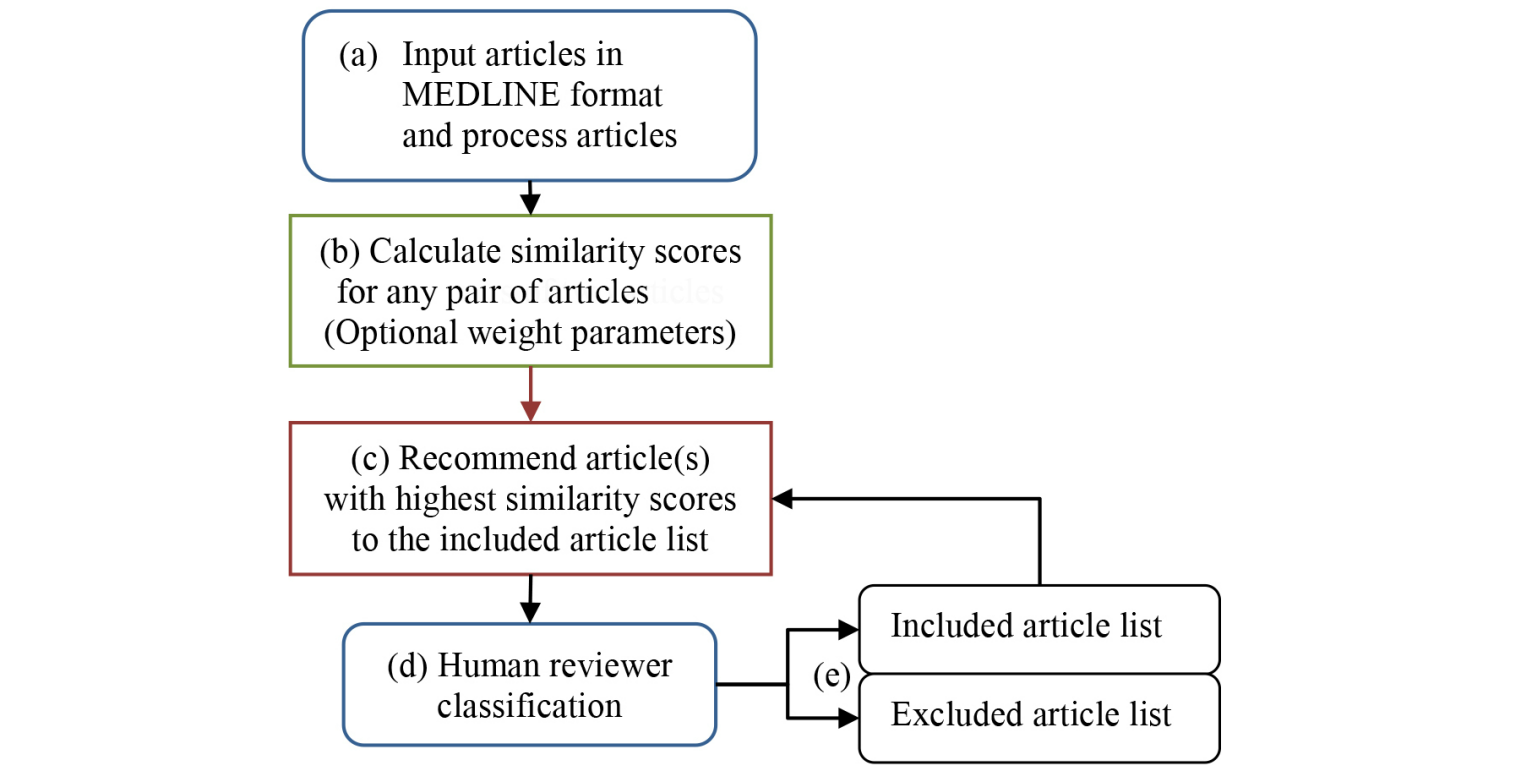
Simulated interactive recommendation process.

Figure 3 illustrates the simulated interactive recommendation process: (a) Process articles and extract data elements; (b) Calculate similarity scores (this will establish a conceptualized article network. Weight parameters are optional); (c) Recommend article(s) with the highest similarity to included articles list (in this simulation, one article is recommended per each round); (d) Human reviewers classify the recommended article as included or excluded (again, in this study, we used completed systematic review reports, which have predetermined decisions to simulate this step); (e) Create and update the included and excluded article list; Steps (c), (d), and (e) repeat until the article screening process is completed.

To evaluate our performance, we used two performance measures: work saved over sampling at 95% recall (WSS95) and F-measure. These measures are commonly used for evaluating similar work [7,12,15]. We also used repeated analysis of variance (ANOVA) and post hoc analysis with Hommel multiple comparison adjustment to further explore statistical evidences. Hommel’s method demonstrated type I error protection with good power and is considered a better approach than Bonferroni or Homl [26,27].

#### Work Saved Over Sampling at 95% Recall

**Figure 4:**
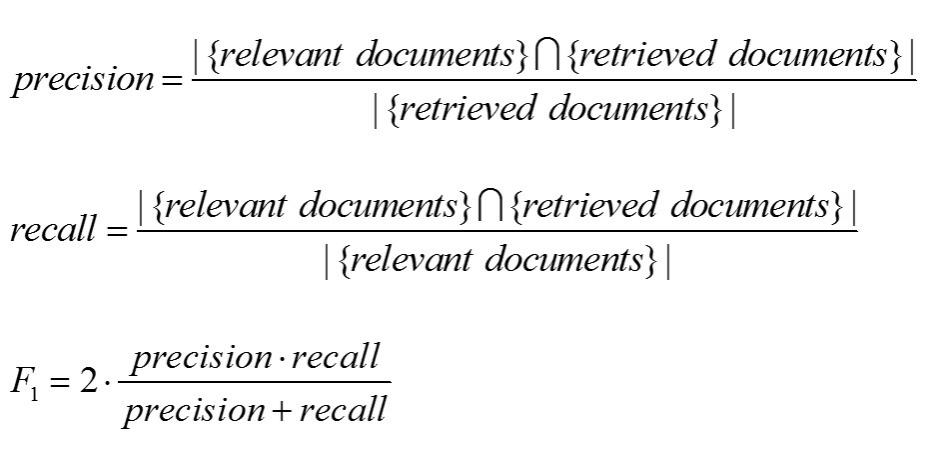
Formulas of precision, recall, and F_1_.

WSS95 is a performance measure first proposed by Cohen [7] to calculate the overall labor saving while maintaining the recall at 95%. This assumes that a recall higher than 0.95 is necessary for a document classification system. Precision should be as high as possible, as long as recall is at least 0.95. WSS95 is calculated with the equations in Figure 4.

TP is the number of true positive (relevant) articles, TN is the number of true negatives (irrelevant) articles, FN is the number of false negative (relevant) articles, and N is the total number of articles in each report.

#### F-Measure

**Figure 5:**
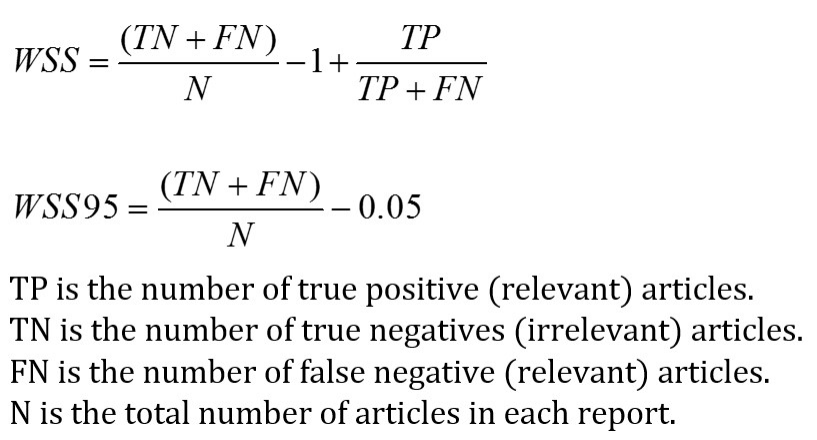
Formulas of WSS and WSS95.

F-measure is a measure of information retrieval accuracy. It considers both precision and recall and commonly combines them into a weighted harmonic mean. When they are weighted equally, the balanced F-measure is also called F_1_, where it reaches its best value at 1 and the worst value at 0. As a general measure of accuracy, F_1_ has been widely used in previous works for the evaluation of classification performance, such as Cohen 2006 [7], Bekhuis 2010 [28], Kastrin 2010 [29], and Frunza 2010 [30]. For our performance evaluation purposes, when we recommend one article each time, the immediate recall, precision, and F_1_ are dynamically changed each time (see Figure 5).

Since F_1_ is dynamically changed over time, we can detect the highest F_1_ from the steepness of the performance curve. That means if the higher F_1_ scores occur during the early stage of the recommendation process (ie, before 50% of articles are screened), we are more likely to save more workload (high accuracy). We use F_1_ to help us evaluate how accurate and how quickly we can make recommendations on the relevant articles.

## Results

### Single Element Performance

The single MEDLINE element performance results are shown in Table 2. TI gets the best average WSS95 performance (34.01%), followed by PT (33.41%), and AB (33.30%). MH has a much lower WSS95 than other elements (25.31%). AU receives 0% workload saved due to the dispersion among articles’ authorship. If there is no authorship similarity between articles, we are not able to recommend relevant articles based solely on AU element. Using PT also brings good performance; we speculate it is a key consideration when conducting system reviews. However, repeated ANOVA shows that the WSS95 performances across TI, AB, PT, and MH are not statistically different (*P*=.079).

**Table 2:** Single element WSS95 performance.

SR report topic	TI	AB	PT	AU	MH
ACE inhibitors	76.49	71.07	33.22	0	47.37
ADHD	80.26	65.10	22.56	0	47.00
Antihistamines	13.55	15.81	32.58	0	2.58
Atypical antipsychotics	17.23	20.54	19.64	0	9.46
Beta blockers	44.74	49.95	43.77	0	28.67
Calcium channel blockers	19.38	16.34	18.64	0	20.94
Estrogens	29.35	29.08	17.93	0	38.59
NSAIDS	63.36	66.67	58.27	0	33.84
Opioids	8.30	9.82	37.23	0	6.48
Oral hypoglycemics	11.73	12.13	22.27	0	7.55
Proton pump inhibitors	43.74	15.60	35.48	0	20.56
Skeletal muscle relaxants	0	36.03	74.68	0	42.85
Statins	25.52	30.17	13.31	0	13.68
Triptans	45.60	42.47	28.17	0	33.23
Urinary incontinence	30.89	18.65	43.43	0	26.91
Average WSS95	34.01	33.30	33.41	0	25.31

Table 3 shows the highest F_1_ performance and corresponding timing during the recommendation process. When performance is good, the highest F_1_ usually occurs during the early stage (discussed in the Methods section). We found that AB and PT gain the best F_1_ (0.3683 and 0.3437, respectively); MH and TI have lower F_1_ scores (0.3116 and 0.3039, respectively). Again, AU gets the worst F_1_, only 0.1365. We also examined the corresponding timing of the highest F_1_. We observed that the best F_1_ value appears when 5% to 20% of articles are screened, which is at the early stage of recommendation. MEDLINE elements with higher F_1_ scores and lower percentages of articles screened indicate high accuracy performance during the early stage of recommendation (eg, AB). We concluded that AB and PT bring the best early stage performance; in other words, the recommendation accuracy of AB and PT in the beginning is better than that of the other elements. However, repeated ANOVA shows that the F_1_ performances across TI, AB, PT, and MH are not statistically different (*P*=.073). Pairwise comparison only finds significant difference between TI and AB (AU is not considered due to its inferior performance).

**Table 3:** Single element F_1_ performance; percentage of articles screened at F_1_.

SR report topic	TI F_1_ (%)	AB F_1_ (%)	PT F_1_ (%)	AU F_1_ (%)	MH F_1_ (%)
ACE inhibitors	0.3444 (4)	0.3121 (4)	0.2182 (<1)	0.1872 (6)	0.2368 (1)
ADHD	0.2885 (10)	0.3824 (6)	0.2963 (<1)	0.0909 (<1)	0.5556 (4)
Antihistamines	0.2593 (12)	0.4000 (3)	0.2759 (<1)	0.1111 (<1)	0.3333 (3)
Atypical antipsychotics	0.3447 (26)	0.4248 (14)	0.4363 (5, 12^a^)	0.0135 (<1)	0.3113 (40)
Beta blockers	0.1972 (1)	0.2710 (5)	0.2105 (<1)	0.0417 (<1)	0.0957 (19)
Calcium channel blockers	0.2026 (10)	0.2672 (11)	0.2662 (15)	0.1261 (9)	0.2579 (2)
Estrogens	0.5140 (36)	0.5612 (29)	0.4937 (18)	0.0244 (<1)	0.5536 (39)
NSAIDS	0.4368 (34)	0.5870 (13)	0.6761 (8)	0.4853 (24)	0.3650 (24)
Opioids	0.2727 (<1)	0.1429 (<1)	0.2222 (<1)	0.1111 (<1)	0.2500 (<1)
Oral hypoglycemics	0.4509 (88)	0.4603 (76)	0.5019 (78)	0.0145 (<1)	0.4527 (53)
Proton pump inhibitors	0.3333 (1)	0.3860 (5)	0.1299 (42)	0.0377 (<1)	0.1775 (25)
Skeletal muscle relaxants	0.1429 (<1)	0.1981 (<1)	0.2286 (2)	0.1429 (<1)	0.2222 (<1)
Statins	0.2278 (6)	0.2479 (1)	0.4019 (4)	0.1484 (12)	0.1563 (1)
Triptans	0.1739 (10)	0.360 (4)	0.2569 (13)	0.0690 (<1)	0.2750 (8)
Urinary incontinence	0.3697 (24)	0.5243 (19)	0.5405 (10)	0.4444 (13)	0.4317 (30)
Average^b^	0.3039 (18)	0.3683 (13)	0.3437 (14)	0.1365 (5)	0.3116 (17)

^a^Both 5% and 12% have F_1_ = 0.4363. The average of 5% and 12% (8.5%) is taken to calculate the average value on the last row of the table.

^b^<1% is considered as 1% for calculating the average percentage.

### Composited Elements Performance

Different MEDLINE elements play different roles in the systematic review process, and their corresponding performance varied greatly as described above. To further explore their predictive abilities, we examined their collaborative performances. In total we examined 22 combinations and chose the top WSS95 performance of 6 combinations (see Table 4). Each of the 6 combination performances has an average of more than 36% WSS95. Table 5 shows the F_1_ performance of the 6 combinations.

We also conducted statistical analysis with repeated ANOVA for the composited elements performance. For WSS95, the results show that there is no statistical difference in WSS95 performance across the 6 combinations (*P*=.332). For F_1_ performance, there is also no statistical significant difference across the 6 combinations (*P*=.069).

In summary, we found that the predictive ability of MEDLINE elements varies according to systematic review topics. Overall, TI and PT have better WSS95 performance on average but are not statistically different. AB has the best average F_1_ scores and is statistically better than TI.

**Table 4:** WSS95 of the top 6 combinations.

SR report topic	TI+AB	TI+AB +MH	TI+AB +PT	TI+AB +AU	TI+AB +PT+AU	TI+AB+MH +PT+AU
ACE inhibitors	76.38	76.85	74.29	75.79	73.70	75.08
ADHD	80.38	79.79	67.92	80.14	67.92	56.17
Antihistamines	16.13	10.65	24.52	16.13	24.52	18.39
Atypical antipsychotics	20.89	14.20	17.95	20.63	17.77	14.38
Beta blockers	60.14	60.09	65.01	60.96	64.72	65.21
Calcium channel blockers	18.23	18.64	17.32	18.39	17.49	22.82
Estrogens	33.42	36.14	22.55	33.97	22.55	29.08
NSAIDS	72.26	75.57	77.35	70.48	76.34	77.86
Opioids	6.01	11.75	8.98	5.95	8.98	12.17
Oral hypoglycemics	11.33	13.12	13.52	11.13	13.52	12.72
Proton pump inhibitors	19.20	21.31	19.65	19.05	19.65	20.11
Skeletal muscle relaxants	41.94	46.44	58.55	41.87	58.49	60.01
Statins	29.10	27.11	27.80	30.96	27.71	26.07
Triptans	48.29	51.71	39.64	50.52	39.79	40.98
Urinary incontinence	12.84	11.01	20.80	12.84	20.80	14.37
Average	36.44	36.96	37.06	36.59	36.93	36.35

**Table 5:** F_1_ of the top 6 combinations.

SR report topic		TI+AB F_1_ (%)		TI+AB+MH F_1_ (%)		TI+AB+PT F_1_ (%)		TI+AB+AU F_1_ (%)	TI+AB+PT+AU F_1_ (%)	TI+AB+MH+PT+AU F_1_ (%)
ACE inhibitors		0.4156 (1)		0.4000 (2)		0.4051 (1)		0.3902 (2)	0.3971 (4)	0.3774 (3)
ADHD		0.4000 (3)		0.4688 (5)		0.5455 (4)		0.4286 (6)	0.5306 (3)	0.5818 (4)
Antihistamines		0.3226 (5)		0.3333 (10)		0.2903 (15)		0.3226 (5)	0.2903 (15)	0.2813 (15)
Atypical antipsychotics		0.4364 (16)		0.4241 (15)		0.4887 (15)		0.4411 (17)	0.4856 (15)	0.4606 (15)
Beta blockers		0.2800 (3)		0.3043 (2)		0.3590 (2)		0.2667 (3)	0.3596 (2)	0.3333 (3)
Calcium channel blockers		0.2335 (8)		0.2620 (11)		0.2804 (9)		0.2323 (8)	0.2816 (9)	0.2995 (9)
Estrogens		0.6000 (30)		0.6237 (29)		0.6047 (25)		0.5979 (31)	0.6118 (24)	0.6171 (26)
NSAIDS		0.6667 (16)		0.6154 (16)		0.6966 (12)		0.6471 (16)	0.6809 (13)	0.6667 (15)
Opioids		0.3000 (0)		0.3158 (<1)		0.3000 (<1)		0.3000 (<1)	0.3000 (<1)	0.3158 (<1)
Oral hypoglycemics		0.4497 (90)		0.4541 (88)		0.4553 (86)		0.4489 (92)	0.4561 (75)	0.4635 (82)
Proton pump inhibitors		0.4384 (7)		0.4737 (5)		0.5172 (5)		0.4552 (7)	0.5455 (5)	0.5079 (6)
Skeletal muscle relaxants		0.2222 (1)		0.2353 (<1)		0.2500 (<1)		0.2222 (1)	0.2500 (<1)	0.2667 (<1)
Statins		0.2994 (2)		0.3281 (1)		0.3382 (1)		0.2959 (2)	0.3358 (2)	0.3465 (1)
Triptans		0.3636 (3)		0.3913 (3)		0.3556 (3)		0.3556 (3)	0.3529 (4)	0.3913 (3)
Urinary incontinence		0.5063 (12)		0.5347 (19)		0.5505 (21)		0.5263 (11)	0.5507 (9)	0.5843 (15)
Average^a^		0.3956 (13)		0.4110 (14)		0.4291 (14)		0.3954 (14)	0.4286 (12)	0.4329 (13)

^a^<1% is considered as 1% for calculating the average percentage.

### Performance Comparison With Existing Literature

Here we also compared our WSS95 performance with existing machine learning model training approaches (we were not able to compare the F_1_ performances as they were not provided). Since TI+AB+PT has the simplest combination and its performance is equivalent or better than others, we chose TI+AB+PT (weight setting = 1:1:1) to compare against existing machine learning model training approaches, including voting perceptron-based automated citation classification system (VP), factorized complement naïve Bayes with weight engineering (FCNB/WE) and support vector machine (SVM) (Table 6).

**Table 6:** WSS95 comparison with the Cohen and Matwin systems across 15 SR topics.

SR report topic	Cohen 2006 [7] (VP^a^)	Cohen 2008 [12] (SVM^b^)	Matwin 2010 [15] (FCNB/WE^c^)	Our study (TI+AB+PT)
ACE inhibitors	56.61	73.30	52.30	74.29
ADHD	67.95	52.60	62.20	67.92
Antihistamines	0	23.60	14.90	24.52
Atypical antipsychotics	14.11	17.00	20.60	17.95
Beta blockers	28.44	46.50	36.70	65.01
Calcium channel blockers	12.21	43.00	23.40	17.32
Estrogens	18.34	41.40	37.50	22.55
NSAIDS	49.67	67.20	52.80	77.35
Opioids	13.32	36.40	55.40	8.98
Oral hypoglycemics	8.96	13.60	8.50	13.52
Proton pump inhibitors	27.68	32.80	22.90	19.65
Skeletal muscle relaxants	0	37.40	26.50	58.55
Statins	24.71	49.10	31.50	27.80
Triptans	3.37	34.60	27.40	39.64
Urinary incontinence	26.14	43.20	29.60	20.80
Average	23.43	40.80	33.50	37.06

^a^VP: voting Perceptron-based automated citation classification system

^b^FCNB/WE: factorized complement naïve Bayes with weight engineering

^c^SVM: support vector machine

The repeated ANOVA test shows significant different across four studies (*P*=.005). The pairwise comparison with Hommel adjustment (Table 7) shows that there is no significant difference between our study and either Cohen 2008 [12] or Matwin 2010 [15] *(P*=.4979, .4979) but is significantly better than Cohen 2006 [7] (*P*=.0475). In summary, our methods provide competitive results to traditional machine learning model training approaches.

We were not able to compare side by side with the Wallace group [13,14] because they used different systematic reviews. Their performance is by far the best among machine learning model training approaches (nearly 50% work reduction without missing any relevant articles) as they incorporate active learning with user interaction, which accepts feedback from users (similar to our Step D in Figure 3) [13,14]. This outcome is predictable as machine learning uses training data to model the classifier. With a large amount of training data, the classifier can perform almost perfectly. However, it is encouraging to us that without using algorithms to formulate a classification model, we are currently able to perform similarly to the model training approaches.

**Table 7:** The P values of pairwise comparison of four studies.

	Cohen 2006	Cohen 2008	Matwin 2010	Our study (TI+AB+PT)
Cohen 2006	—	0.0012	0.0433	0.0475
Cohen 2008	0.0012	—	0.0649	0.4979
Matwin 2010	0.0433	0.0649	—	0.4979
Our study	0.0475	0.4979	0.4979	—

### Performance With Weight Parameters

Since different systematic reviews have diverse scopes (for example, one may require sufficient study information from an AB while another may have strict criteria on PT), we were interested in whether different weight parameters would alter the performance. We conducted experiments on different weight settings (eg, TI:PT:AU=3:1:2, TI:PT:AU=2:2:1, TI:PT:AU=3:2:1). The results revealed that when one element’s weight was increased to achieve a higher performance for some reports, some other reports would have performance degradation. Overall, we could not find a universal weight setting that benefited all reports. This may be explained in part by the diverse scopes captured in different systematic reviews. In addition, although some weighted combinations bring better global performance (ie, average WSS95 among 15 reports), the enhancement from the baseline (elements in the combination are equally weighted) is limited. For example, consider the combination of TI+PT+AU, the baseline performance (TI:PT:AU=1:1:1) evaluated by average WSS95 is 35.45%, while the performance of its weighted one (TI:PT:AU=3:1:2) (37.30%) gains less than 2%. There is not much improvement with weighted parameters.

### Interpreting the Inconsistency of WSS95 and F_1_

**Figure 6:**
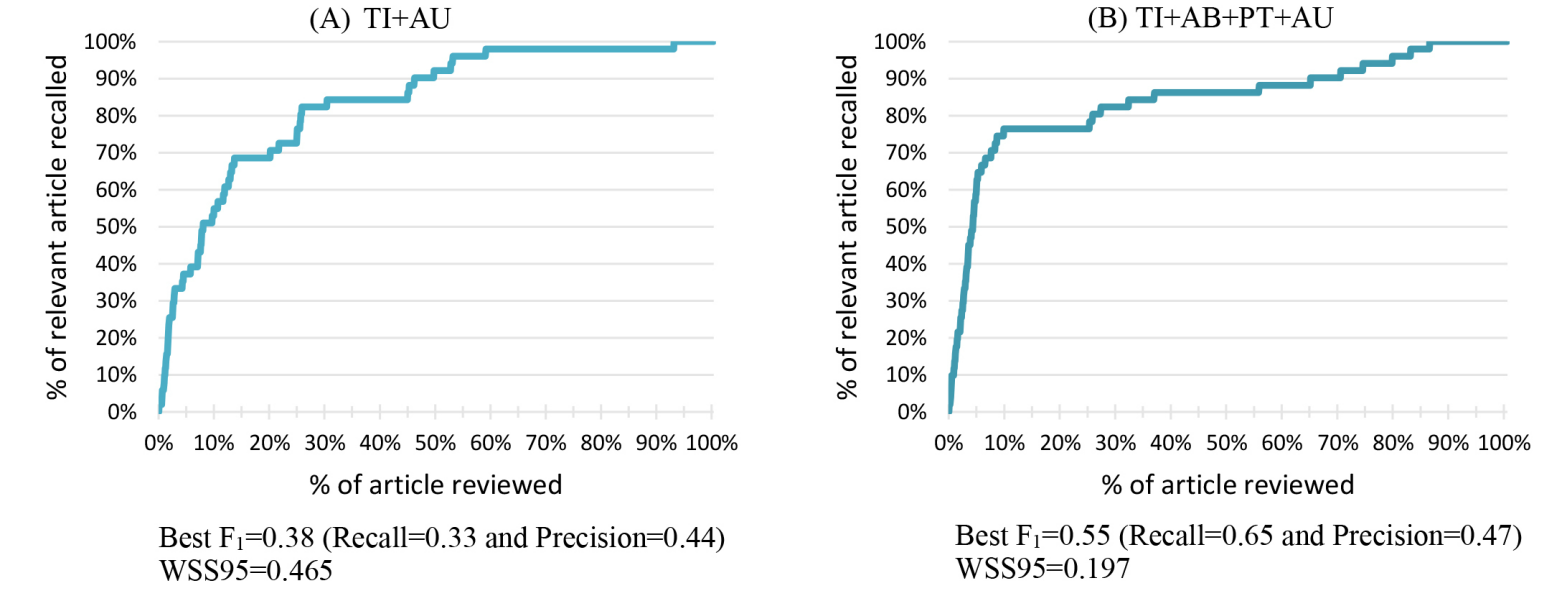
Proton Pump Inhibitors recall performance during the recommendation process using two different element combinations.

During our experiments, we also discovered inconsistencies in performance with respect to WSS95 and F_1_. For example, some combinations had high F_1_ performance with low WSS95 and vice versa. We examined the recall performance during the entire recommendation process. Figure 6 presents the performance curves of the Proton Pump Inhibitors systematic review with two different element combinations, TI+AU (Figure 6A) and TI+AB+PT+AU (Figure 6B) (all equally weighted). The x-axis represents the percentage of articles screened (or recommended); the y-axis represents the recall rate. From the Figure 6, we see that in the early screening stage (5% of articles screened), curve B (recall of 70%) is steeper than curve A (recall of 40%). This also shows in their F_1_ scores: the highest F_1_ scores of curve A and B are 0.3778 and 0.5455, respectively, during the early screening stage. However, at the later stage, curve A reaches the recall of 100% faster than curve B after screening 60% of articles (WSS95 scores of curve A and B are 46.51% and 19.65%, respectively). In summary, current performance measures using WSS95, area under the curve, precision, and recall could not reflect the performance over time. Some elements may accelerate the performance in the beginning of the recommendation (screening) process. Using multiple performance measures and especially including the highest F_1_ at a certain time point can better help us recognize the strength and weakness of different elements during the entire screening process

## Discussion

### Customizable Weight Parameters May Enhance Performance More Efficiently

Due to the fact that different systematic reviews have different review scopes, we could not identify one universal weight setting which could be successfully applied to every systematic review. A similar idea was mentioned in Matwin’s work [15], where weight parameters (or weight multipliers) were tunable and being modified with regards to different systematic reviews. While different systematic reviews should have different weight multiplier values, we also agree that the process of computing such a value for every systematic review would be very time consuming [15]. Therefore, instead of finding the best weight parameters for each systematic review, flexible, customizable weight parameters for human reviewers based on their systematic review scopes and screening priority would be more useful and practical. Without adjusting weight parameters, our average performance is higher than the FCNB/WE approach [15] (Table 6). It is likely that we could improve even further when adjustable weight parameters are provided to human reviewers.

### Moving Toward an Efficient and Generalizable Approach

Currently the work of biomedical text classification for the purpose of reducing systematic review workload has mainly used machine learning model training approaches. Naïve Bayes and SVM are two widely applied machine learning algorithms. Although these machine learning approaches provide excellent performance in text classification on specific systematic review topics, it is a challenge to apply the existing machine learning algorithms to other new systematic review topics. It could be time consuming to construct training models as well [15]. In addition, the implementation of machine learning approaches usually requires an understanding of the algorithm. For example, operators need to choose a kernel or tune the setting of parameters for the SVM algorithm. Thus, it is difficult to apply the approach to a new systematic review topic without a well-trained classification model or without significant machine learning knowledge. Other approaches, such as text mining or statistical approaches, were also studied to facilitate the systematic review process [29,31], but they also rely heavily on prior decisions to find key terms to differentiate between the relevant and irrelevant classes, which is very similar to supervised machine learning.

Overcoming the limitations mentioned above, we provided a generalizable approach which can be easily deployed to facilitate any systematic review. Also, because we established an article network providing similarity relationship between articles, the iterative interactive recommendation process takes almost no time. Currently, our processing time to construct an article network takes from several seconds to several minutes for 300 to 3500 articles, but the recommendation step is real-time. This processing time is reasonable considering the non-trivial steps of building article networks. To be specific, this is polynomial time processing, not linear time processing. In our study, the backend programs for the computation of similarity matrixes are written in C/C++, which is the most efficient approach from the perspective of computer architecture and compiler. We also plan to improve the time responses for larger systematic reviews that may contain ten thousand articles or more. Most importantly, our approach can be applied to any systematic review topic and nontechnical human reviewers can use it with ease.

### Study Limitation

This study only uses 15 DERP reports for evaluation. Although it is our assumption that our approach will be applicable globally, datasets from other systematic review teams are needed to further demonstrate our hypothesis. Our future plans include collaborating with other systematic review teams.

### Future Direction

As we have discussed in the Methods section, different article elements have different predictive abilities regarding the evaluation scheme of WSS95 or F_1_ score. With a better F_1_ score and a lower WSS95, combinations containing AB or MH are more likely to elicit good performance in the beginning but have difficulty reaching 100% recall. On the other hand, although the combination of TI, PT, and AU can reach a better overall workload saved, the recall rises slowly (low accuracy) in the beginning of the recommendation process. This inspires us to utilize multiple types of weight settings and take advantage of different article element strengths during different recommendation phases (early-, mid-, and late-phases). We plan to implement automatic detection and adjustment when information from elements has been exhausted, which indicates the time to alter the combination of elements and weight parameters. For instance, when a series of N recommended articles is classified as excluded by human reviewers, we take it as a signal for adjustment as the current setting can no longer provide a good recommendation. Another example is to first apply the combination of AB and MH, as they provide high accuracy in the early recommendation stage, and then automatically adjust to the combination of TI, PT, and AU in the subsequent phase. Further research is also necessary to investigate proper adjustments of weight parameters under different conditions.

In the near future, we will also provide visualized article networks where relationships between articles could be intuitively represented and comprehended by humans. Network-based analysis will be conducted and network metrics like graph diameter, centrality, and module classes (by communication detection) will be reported. Such visualizations have the potential to enable the identification of clusters of articles and knowledge gaps in a targeted area. Lower density in such visualizations of the network could also indicate fewer related articles published or vice versa.

### Conclusions

We demonstrated a new approach to assist the systematic review article screening process. We established article networks based on article similarity that facilitate the process of interactive article recommendation. We calculated article similarities using MEDLINE elements and examined the predictive ability of the MEDLINE element(s). We found that TI and PT have the best WSS95 performance, and AB and PT provide the best F_1_ scores during the early stage of the recommendation process. However, no statistical difference was found.

Using our approach, we are able to achieve an average of 37% WSS95 with equally weighted combination of TI, AB, and PT. The statistical analysis also demonstrated that it is competitive with existing approaches. Based on findings and lessons learned from this study, we are currently deploying the approach into a prototype public online system, ArticleNet, to assist the article screening process.

## Abbreviations

AB: abstract
ANOVA: analysis over variance
AU: author
DERP: Drug Effectiveness Review Project
EBM: evidence-based medicine
EX: excluded
F_1_: F-measure
FCNB/WE: factorized complement naïve Bayes with weight engineering
IN: included
MH: MeSH, or medical subject heading
PMID: PubMed Indentifier
PT: publication type
SVM: support vector machine
TI: title
VP: voting perceptron-based automated citation classification system
WSS95: worked saved over sampling at 95% recall
